# Associations of blood mitochondrial DNA copy number with social-demographics and cancer risk: results from the Mano-A-Mano Mexican American Cohort

**DOI:** 10.18632/oncotarget.25321

**Published:** 2018-05-22

**Authors:** Hua Zhao, David Chang, Yuanqing Ye, Jie Shen, Wong-Ho Chow, Xifeng Wu

**Affiliations:** ^1^ Department of Epidemiology, The University of Texas MD Anderson Cancer Center, Houston, 77030 TX, USA

**Keywords:** mitochondrial DNA copy number, cancer risk, lifestyle factors, social context

## Abstract

The relationship between blood mitochondrial DNA (mtDNA) copy number and subsequent cancer risk has been investigated previously. However, such association has never been examined in Mexican Americans. In the current study, we examined association between social-demographic factors and blood mtDNA copy number, as well as longitudinal relationship between cancer and mtDNA copy number, among 10,802 Mexican Americans in the Mano-A-Mano Mexican American Cohort. Overall, mtDNA copy number was statistically significantly higher among participants who developed cancer during the study period than among cancer-free controls (0.17 vs 0.13, *P* = 0.007). Among cancer-free control participants, mtDNA copy number significantly differed by social-demographic characteristics. However, there was a large degree of heterogeneity in these effects across the mtDNA copy number distribution. In the longitudinal analysis, we observed that higher mtDNA copy number was positively associated with increased risk of all cancer types (adjusted hazard ratio [HR], 1.13; 95% confidence interval [CI], 1.09–1.17). Participants with mtDNA copy number in the fourth (highest) quartile had a higher risk of all cancer (adjusted HR, 2.12; 95% CI, 1.65–2.73) than did participants in the first (lowest) quartile. In summary, our results in Mexican Americans support an association between increased mtDNA copy number and cancer risk. Our results also suggest that mtDNA copy number may be influenced by social and demographic factors.

## INTRODUCTION

Mitochondrial DNA (mtDNA) is highly vulnerable to oxidative damage because it lacks protection from introns and histones and has less efficient DNA repair mechanisms than nuclear DNA does [1, 2]. The number of copies of mtDNA varies widely in humans, ranging from 100 to 10,000 copies per cell, depending on the cell type [3]. Each type of cell or tissue has a fairly constant mtDNA copy number [4]. Precisely how mtDNA copy number is maintained remains poorly understood, but the mechanism probably involves mitochondrial function, energy metabolism, and oxidative damage.

Over the past decade, molecular epidemiologic studies have examined the relationships between mtDNA copy number in leukocytes or whole blood and the risk of several kinds of cancer [5–16]. Positive, inverse, and null associations were reported in those studies. However, whether mtDNA copy number is associated with cancer risk has never been examined in any prospective cohort of adult Mexican Americans.

Indeed, understanding the role of blood mtDNA in cancer risk is particularly important for Mexican Americans, one of the fastest-growing populations in the United States [17]. Three-quarters of Mexican Americans are either overweight or obese [18, 19]; mtDNA copy number may be a predictive marker of metabolic disturbances, including obesity [20]. Mexican Americans also have a high burden of age-related chronic diseases, such as type 2 diabetes and cardiovascular disease [21–24], both of which are associated with mitochondrial dysfunction. Finally, Mexican Americans experience distinctive psychological, somatic, and social stressors associated with immigration and acculturation [25, 26]. These stressors may affect glucocorticoids and correspondingly influence mitochondria by altering expression of mitochondrial genes and nuclear genes that influence mitochondrial function and copy number [27, 28].

In the current study, our primary hypothesis was that blood mtDNA copy number is associated with overall cancer risk among Mexican Americans. We further hypothesized that blood mtDNA copy number was modified by social, demographic, and lifestyle characteristics at baseline. To test the hypotheses, we measured the relative mtDNA copy number in bloods from 10,802 participants in the large ongoing prospective Mexican American Mano a Mano Cohort Study. We determined the relationship of mtDNA copy number with social, demographic, and lifestyle characteristics at baseline and with the development of cancer subsequently.

## RESULTS

### Study cohort characteristics

**Figure 1:**
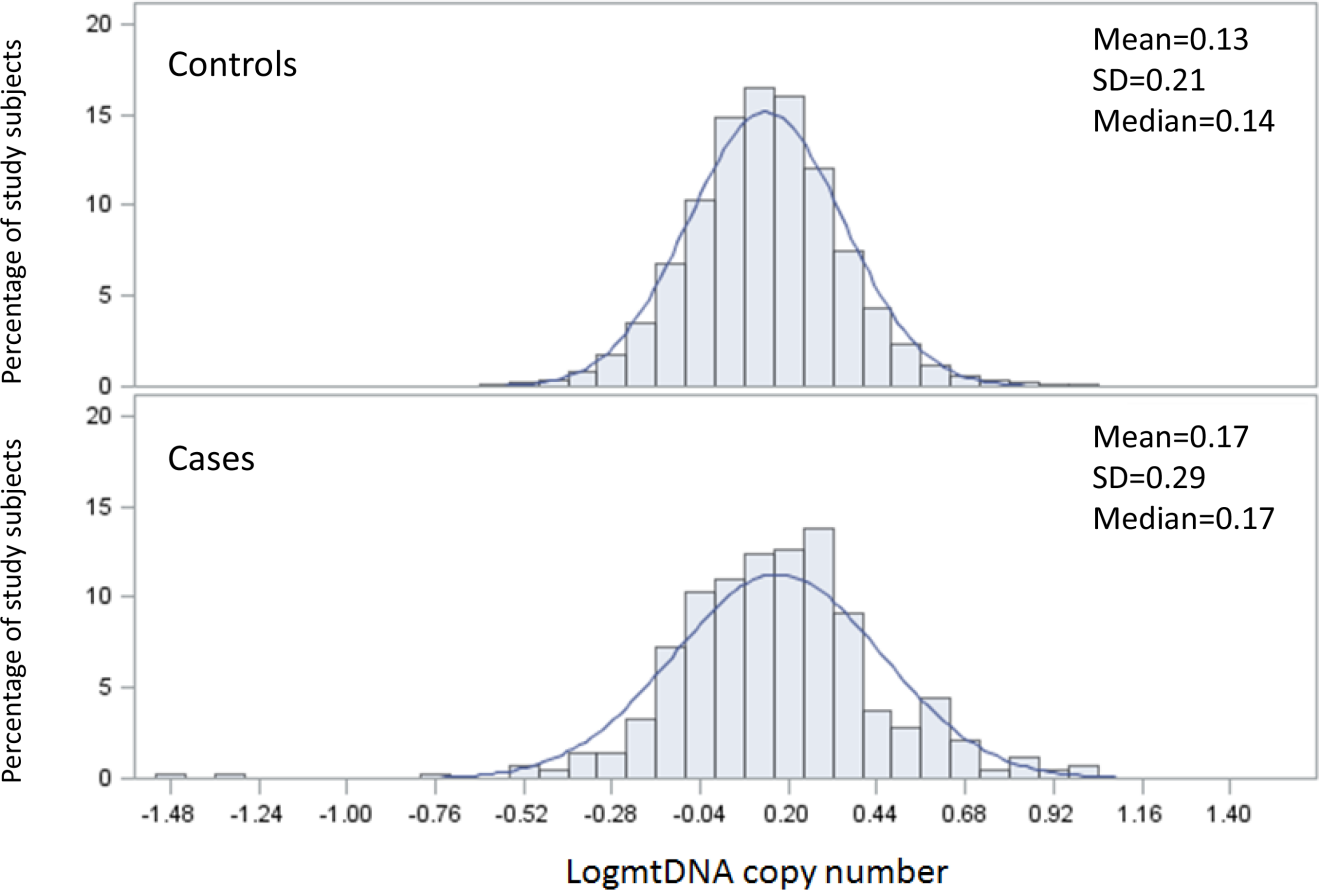
The distribution of mtDNA copy number by case control status

A total of 10,802 participants were included in the analysis. Among them, 429 developed cancer during the follow-up period. The mean mtDNA copy number was significantly higher among participants who developed cancer than in the cancer-free control group (0.17 vs. 0.13, *P* = 0.007) (Figure 1).

### Factors associated with differences in mtDNA copy number

We first evaluated whether mtDNA copy number differed by participant characteristics in the 10,373 cancer-free control participants (Table 1). The most significant differences in mtDNA copy number were observed in relation to age, sex, birthplace, and BMI. As age increased, mean mtDNA copy number significantly decreased (*P* < 0.001). Women and participants born in Mexico had significantly higher mean mtDNA copy numbers than did men and US-born participants (both *P* < 0.001). Mean mtDNA copy number was also significantly higher among overweight participants than among participants who were underweight, normal weight, or obese (*P* < 0.001). In addition, we found that mean mtDNA copy number significantly differed by marital status (*P* = 0.024), education level (*P* = 0.024), history of alcohol use (*P* = 0.021), and physical activity level (*P* = 0.016), but not by smoking status (*P* = 0.054) or time per day spent on sitting (*P* = 0.198).

**Table 1:** Log-transformed mitochondrial DNA (mtDNA) copy number by physical and sociodemographic characteristics among 10,373 cancer-free control participants

Variable	*N* (%)	Mean mtDNA copy number (SD)	*P* value
Overall	10,373 (100)	0.13 (0.21)	
Age at enrollment, years (*N* = 10,373)			
21–30	2279 (21.97)	0.15 (0.20)	
31–40	3431 (33.08)	0.15 (0.21)	
41–50	2258 (21.77)	0.14 (0.21)	
>50	2405 (23.19)	0.11 (0.22)	< 0.001
Sex (*N* = 10,373)			
Male	2062 (19.88)	0.12 (0.21)	
Female	8311 (80.12)	0.14 (0.21)	< 0.001
Marital status (*N* = 10,370)			
Married/living together	8126 (78.36)	0.14 (0.21)	
Other	2234 (21.54)	0.13 (0.22)	0.024
Education (*N* = 10,367)			
Less than high school	6097 (58.81)	0.13 (0.22)	
High school graduate	2323 (22.41)	0.15 (0.20)	
College	1947 (18.78)	0.14 (0.21)	0.024
Place of birth (*N* = 10,373)			
Mexico	7813 (75.32)	0.14 (0.21)	
US	2560 (24.68)	0.12 (0.21)	< 0.001
BMI (*N* = 10,349)			
Underweight/normal weight	1501 (14.50)	0.13 (0.21)	
Overweight	3423 (33.08)	0.15 (0.22)	
Obese	5425 (52.42)	0.13 (0.21)	< 0.001
Smoking status (*N* = 10,371)			
Never	7571 (73.00)	0.14 (0.21)	
Former	1526 (14.71)	0.13 (0.21)	
Current	1274 (12.28)	0.13 (0.22)	0.054
Alcohol drinking (*N* = 10,351)			
Never	6993 (67.56)	0.14 (0.21)	
Former	1015 (9.81)	0.12 (0.22)	
Current	2343 (22.64)	0.14 (0.20)	0.021
Physical activity (*N* = 10,283)			
Low	7471 (72.65)	0.13 (0.21)	
Medium + High	2812 (27.35)	0.15 (0.20)	0.016
Sitting, hours per day (*N* = 10,347)			
<2	2967 (28.67)	0.14 (0.21)	
2–4	4234 (40.92)	0.14 (0.21)	
4–6	1952 (18.87)	0.14 (0.21)	
>6 hours	1194 (11.54)	0.13 (0.21)	0.198

Abbreviations: SD, standard deviation; BMI, body mass index.

### Quantile analyses

To further understand the relationship between mtDNA copy number and selected characteristics in the control group, we performed multivariate unconditional quantile regression analyses. All variables that displayed significant differences shown in Table 1, specifically, age, sex, marital status, education level, birthplace, BMI, alcohol drinking status, and physical activity level, were included in this analysis. Table 2 shows the estimated quantile regression coefficients for 5 selected quantiles (10th, 25th, 50th, 75th, and 90th percentiles) in the control group. Significant inverse relationships were consistently observed between age and mtDNA copy number in all 5 percentiles (all *P* < 0.01), though the associations became weaker as the percentile increased. Women and participants born in Mexico had higher mtDNA copy numbers than did men and US-born participants, and these correlations were statistically significant at the 25th, 50th, 75th, and 90th percentiles of mtDNA copy number.

**Table 2:** Correlations between participant characteristics and log-transformed mitochondrial DNA (mtDNA) copy number by mtDNA copy number quantile rank in cancer-free controls

Variable	Percentile 10	Percentile 25	Percentile 50	Percentile 75	Percentile 90
Age	–0.0017^**^	–0.0014^**^	–0.0013^**^	–0.0013^**^	–0.0012^**^
Sex					
Female vs. Male	0.0057	0.0149^*^	0.0228^**^	0.0301^**^	0.0268^*^
Marital Status					
Other vs. Married/living together	–0.0122	–0.0113	–0.0008	0.0047	–0.0006
Education					
College	Reference				
Less than high school	–0.0038	–0.0054	–0.0008	–0.0014	0.0012
High school graduate	0.0217^*^	0.0091	0.0049	0.002	0.0007
Place of birth					
US	Reference				
Mexico	0.0086	0.0208^**^	0.0172^**^	0.0183^**^	0.0270^**^
BMI					
Obese	Reference				
Underweight/normal weight	–0.0194^*^	–0.0248^**^	–0.0146^*^	–0.0023	0.0024
Overweight	–0.001	0.0066	0.0181^**^	0.0208^**^	0.0233^**^
Alcohol drinking					
Never	Reference				
Former	–0.0333^**^	-0.0116	0.0045	–0.0007	0.0026
Current	–0.0074	0.0038	0.0049	0.0076	0.0300^*^
Physical activity					
Medium/High vs Low	–0.0308^**^	–0.0148^**^	–0.0059	–0.0101	0.3373

Abbreviation: BMI, body mass index. ^*^5% significance; ^**^1% significance.

We also found that BMI was associated with mtDNA copy number. Using obese participants as the reference group, we found that at the 10th, 25th, and 50th percentiles, participants who were underweight or normal weight had significantly lower mtDNA copy numbers. Indeed, we observed a trend toward a weaker or even an inverse correlation as the mtDNA copy number percentile increased. However, overweight participants in the 50th, 75th, and 90th percentiles had significantly higher mtDNA copy numbers than did their counterparts in the obese group; the correlation between overweight and higher mtDNA copy number became stronger as the mtDNA copy number percentile increased. Regarding physical activity, participants with medium and high levels of physical activity had higher mtDNA copy numbers than did those with low levels of physical activity, except for participants in the 90th percentile of mtDNA copy number. These associations were significant at the 25th and 50th percentiles. In addition, we observed a weaker and even an inverse association between mtDNA copy number and physical activity as the copy number percentile rank increased.

### Association of mtDNA copy number with acculturation and socioeconomic status

Next, we investigated the relationships between mtDNA copy number and variables measuring acculturation, immigration, and socioeconomic status among cancer-free participants. To measure acculturation, we included a language acculturation score and the preference for Mexican or US food, friends, social activity, heritage, and holidays. For participants who were born in Mexico, the number of years living in the United States and the age at immigration were also included in the analysis. Measures of socioeconomic status included income, home ownership, car ownership, and health insurance status. In addition, we included age, sex, and birthplace in the analysis. Among cancer-free study subjects, those who showed a weaker preference for Mexican friends had significantly higher mtDNA copy numbers (*P* = 0.011). However, car ownership was significantly inversely correlated with mtDNA copy number (*P* = 0.036). We next stratified the study cohort by birthplace. We found that among participants born in Mexico, a lower preference for Mexican friends and fewer years of living in the United States were significantly positively correlated with mtDNA copy number (*P* = 0.034 and 0.010, respectively). Having health insurance was marginally positively correlated with mtDNA copy number for participants born in Mexico (*P* = 0.080). For participants born in the United States, higher levels of language acculturation and lower preference for celebrating Mexican holidays were significantly positively correlated with mtDNA copy number (*P* = 0.024 and 0.008, respectively). In addition, a marginal inverse relationship was observed between social and car ownership and mtDNA copy number (*P* = 0.061 and 0.064, respectively).

### Mitochondrial DNA copy number and cancer risk

We next examined the relationship between mtDNA copy number and cancer risk (Table 3). In the univariate analysis, we found that elevated mtDNA copy number (as a continuous variable) was associated with an increased risk of all cancers (HR, 1.10; 95% CI, 1.05–1.14). We also identified several other cancer risk factors, including age, sex (male vs female), marital status (other vs married/living as married), birthplace (US-born vs Mexico-born), BMI (obese vs underweight/normal weight), smoking status (former and current vs never smokers), and alcohol drinking status (current vs never alcohol drinking). In the subsequent multivariate analysis, we included all identified cancer risk factors and mtDNA copy number. The association between elevated mtDNA copy number and cancer risk was slightly stronger than in the univariate analysis (HR, 1.13; 95% CI, 1.09–1.17). In addition, age, birthplace, BMI, and smoking status remained significantly associated with increased risk of all cancers.

**Table 3:** Univariate and multivariate analyses of associations between characteristics, log-transformed mtDNA copy number, and cancer risk

Variable	Cases	Controls	HR (95% CI)	*P* value	Adj. HR (95% CI)	*P* value
	*N* = 429 (%)	*N* = 10,373 (%)				
Mean age at enrollment, years (SD)	53.8 (14.2)	41.3 (13.0)	1.06 (1.05–1.06)	<0.001	**1.06 (1.05–1.06)**	**<0.001**
Sex						
Female	326 (75.99)	8311 (80.12)	Reference			
Male	103 (24.01)	2062 (19.88)	1.25 (1.00–1.56)	0.048		
Marital status						
Married/living as married	311 (72.49)	8126 (78.44)	Reference			
Other	118 (27.51)	2234 (21.56)	1.41 (1.14–1.74)	0.004		
Education						
College	74 (17.25)	1947 (18.78)	Reference			
Less than high school	272 (63.40)	6097 (58.81)	1.11 (0.87–1.42)	0.397		
High school graduate	83 (19.35)	2323 (22.41)	1.22 (0.94–1.58)	0.132		
Place of birth						
US	173 (40.33)	2560 (24.68)	Reference		Reference	
Mexico	256 (59.67)	7813 (75.32)	0.52 (0.43–0.63)	<0.001	**0.74 (0.60–0.90)**	**0.003**
BMI						
Underweight/Normal weight	50 (11.66)	1501 (14.50)	Reference		Reference	
Overweight	146 (34.03)	3423 (33.08)	1.37 (0.99–1.90)	0.055	1.25 (0.90–1.72)	0.184
Obese	233 (54.31)	5425 (52.42)	1.49 (1.09–2.03)	0.012	**1.40 (1.03–1.91)**	**0.033**
Smoking status						
Never	255 (59.44)	7571 (73.00)	Reference		Reference	
Former	94 (21.91)	1526 (14.71)	1.84 (1.43–2.37)	<0.001	1.23 (0.96–1.56)	0.096
Current	80 (18.65)	1274 (12.28)	1.87 (1.47–2.36)	<0.001	**1.86 (1.44–2.40)**	**<0.001**
Alcohol drinking						
Never	270 (62.94)	6993 (67.56)	Reference			
Former	71 (16.55)	1015 (9.81)	0.96 (0.76–1.22)	0.753		
Current	88 (20.51)	2343 (22.64)	1.75 (1.35–2.28)	<0.001		
Physical activity						
Low	296 (77.28)	7763 (73.13)	Reference			
Medium/High	87 (22.72)	2852 (26.87)	0.83 (0.66–1.04)	0.096		
Sitting time per day, h						
<2	115 (26.81)	2967 (28.67)	reference			
2–4	175 (40.79)	4234 (40.92)	1.04 (0.82–1.31)	0.763		
4–6	76 (17.72)	1952 (18.87)	1.01 (0.76–1.35)	0.945		
6+ hours	63 (14.69)	1194 (11.54)	1.29 (0.95–1.76)	0.105		
mtDNA copy number						
Continuous (unit = 0.1)	427 (100)	10,373 (100)	1.10 (1.05–1.14)	<0.001	**1.13 (1.09–1.17)**	**<0.001**

To further assess the relationship between mtDNA copy number and risk of cancer, we stratified the study subjects using the median mtDNA copy number in the non-cancer group (0.13) as the cutoff point. We found that those with an mtDNA copy number of 0.13 or higher had a higher risk of all cancers than those with a lower mtDNA copy number (HR, 1.71; 95% CI, 1.41–2.07) (Table 4). When mtDNA was grouped into quartiles, cancer risk increased significantly with increasing quartile in a dose-response manner (*P* < 0.001), from 1.02 in the second quartile to 1.32 and 2.12 in the third and fourth quartile compared to those in the first quartile (i.e., those with the lowest mtDNA copy number). We also examined the impact of the time between cancer diagnosis and whole blood collection on the relationship between mtDNA copy number and cancer risk, using 5 years as the cutoff point (Table 4). The association between mtDNA copy number and cancer risk was stronger for participants whose cancer was diagnosed within 5 years of blood collection than for those whose cancer was diagnosed later, though it was significant in both groups.

**Table 4:** Multivariate analysis of association between log-transformed mtDNA copy number and cancer risk

All cancers				
logmtDNA	Cases, *N* (%)	Controls, *N* (%)	Adj, HR (95% CI)^*^	*P* value
Continuous (unit = 0.1)	427 (100)	10,373 (100)	1.13 (1.09–1.17)	<0.001
By median				
<0.13	188 (44.03)	5081 (48.98)	Reference	
≥0.13	239 (55.97)	5292 (51.02)	1.71 (1.41–2.07)	<0.001
By quartile				
1st	107 (25.06)	2468 (23.79)	Reference	
2nd	81 (18.97)	2613 (25.19)	1.02 (0.76–1.36)	0.906
3rd	90 (21.08)	2643 (25.48)	1.32 (0.99–1.75)	0.055
4th	149 (34.89)	2649 (25.54)	2.12 (1.65–2.73)	<0.001
*P* for trend				<0.001

All cancers: cancer diagnosed ≤5 years after whole blood collection				
logmtDNA	Cases, *N* (%)	Controls, *N* (%)	Adj. HR (95% CI)^*^	*P* value
Continuous (unit = 0.1)	188 (100)	3420 (100)	1.21 (1.13–1.29)	<0.001
By median				
<0.13	67 (35.64)	1635 (47.81)	Reference	
≥0.13	121 (64.36)	1785 (52.19)	2.18 (1.61–2.96)	<0.001
By quartile				
1st	34 (18.09)	707 (20.67)	Reference	
2nd	33 (17.55)	928 (27.13)	0.96 (0.60–1.55)	0.873
3rd	53 (28.19)	980 (28.65)	1.64 (1.06–2.54)	0.026
4th	68 (36.17)	805 (23.54)	2.83 (1.86–4.32)	<0.001
*P* for trend				<0.001

All cancers: cancer diagnosed >5 years after whole blood collection				
Continuous (unit = 0.1)	205 (100)	6572 (100)	1.09 (1.03–1.15)	0.002
By median				
<0.13	105 (51.22)	3299 (50.20)	Reference	
≥0.13	100 (48.78)	3273 (49.80)	1.44 (1.09–1.90)	0.010
1st	68 (33.17)	1707 (25.97)	Reference	
2nd	37 (18.05)	1592 (24.22)	0.80 (0.53–1.19)	0.263
3rd	32 (15.61)	1544 (23.49)	0.99 (0.58–1.34)	0.549
4th	68 (33.17)	1729 (26.31)	1.74 (1.23–2.44)	0.002
*P* for trend				0.003

Breast cancer only				
Continuous (unit=0.1)	102 (100)	8311 (100)	1.23 (1.14–1.32)	<0.001
By median				
<0.13	33 (32.04)	3988 (47.98)	Reference	
≥0.13	69 (67.96)	4323 (52.02)	2.63 (1.73–4.00)	<0.001
1st	19 (18.63)	1939 (23.33)	Reference	
2nd	14 (13.73)	2049 (24.65)	0.92 (0.46–1.83)	0.808
3rd	24 (23.53)	2131 (25.64)	1.80 (0.98–3.30)	0.058
4th	45 (44.12)	2192 (26.37)	3.25 (1.89–5.60)	<0.001
*P* for trend				<0.001

^*^Adjusted by age, birth place, BMI category, and smoking status.

Finally, we conducted an analysis focused on breast cancer, by far the most common cancer in this cohort. Breast cancer risk also increased with mtDNA copy number (as a continuous variable) (HR, 1.23; 95% CI, 1.14–1.32) but with a slightly higher HR than that estimated for all cancer. With the cohort stratified by the median mtDNA copy number in the control group, the group with high mtDNA copy number had a higher risk of breast cancer than did the group with low mtDNA copy number (HR, 2.63; 95% CI, 1.74–4.00). Participants in the fourth mtDNA copy number quartile had a higher risk of breast cancer than did those in the first quartile (HR, 3.25; 95% CI, 1.89–5.60).

## DISCUSSION

To our knowledge, this is the first study to evaluate the relationship between mtDNA copy number and cancer risk among Mexican Americans. Overall, mtDNA copy number was statistically significantly higher among participants with cancer than in the cancer-free cohort group. Among non-cancer participants, mtDNA copy number significantly differed by age, sex, birthplace, BMI, physical activity level, and several variables measuring acculturation, immigration, and socioeconomic status. Risk assessment revealed a statistically significant positive association between high mtDNA copy number and risk of all cancers. These associations were even more pronounced when cases of breast cancer were considered separately and among participants who developed cancer during the first 5 years of follow-up.

Although all our study participants are Mexican Americans, given the critical role of mitochondria in the biological system, we don’t expect to see large racial or ethnic differences in comparison of the results from this study to those conducted in other race or ethnic groups. In fact, the observed relationship between mtDNA copy number and breast cancer risk is similar with other studies conducted in other race and ethnic groups, including Caucasian, Chinese, etc [5, 6, 29, 30]. In addition, the impact of blood drawn on the association observed in this study is also reported in previous studies [5, 11].

However, two recent meta-analyses found no significant association between mtDNA copy number and all-cancer risk [31, 32]. The discrepancy between the results of those studies and ours may be attributable to the heterogeneity of the cancer sites in the study samples. Those meta-analyses demonstrated that the relationship between mtDNA copy number and cancer risk markedly varied by cancer site, with the most consistent relationships observed in lymphoma and breast cancer. In our study, nearly a quarter of the cancer cases were breast cancer, substantially higher than in the 2 meta-analyses. We observed a significant positive association between mtDNA copy number and breast cancer risk. This observation is consistent with other reports from the literature [5, 6, 29, 30]. The discrepancy may also come from the difference in study design. The meta-analyses include both case-control and nested case-control studies, and our study is a prospective cohort study, which provide us the ability to determine temporal exposure in relation to disease and evaluate the possibility of reverse causation. Interestingly, studies have shown that changes in mtDNA content might be a genetic event in the progression of carcinogenesis. For example, mtDNA copy number was higher in early-stage ovarian tumors, where late-stage tumors had lower mtDNA copy number [33]. And in gastric cancer, mtDNA instability may play a role in early events leading to cancer progression [34]. As the associations we observe are more pronounced among cases occurring earlier during follow-up, our results suggest increased levels of mtDNA copy number may be an early biomarker of subclinical cancer in Mexican Americans. Our observations are consistent with those of 3 previous reports [5, 11]. Clearly, more studies are needed to further assess the biological significance of mtDNA copy number difference between the cases and controls. Additionally, our study showed that differences in the social-demographic characteristics, acculturation and immigration experiences, and health behaviors of the study populations may also contribute to the discrepancy.

We observed blood mtDNA copy number was inversed associated with age among the non-cancer participants. This finding is consistent with the results of several previous studies of various organ tissues [29, 30, 35–39]. We also observed a sex difference in mtDNA copy number in the control group. Similar sex differences were reported in previous studies [12, 16, 40]. Our study is the first to report differences in mtDNA copy number by birthplace, with Mexico-born participants having higher mtDNA copy numbers than US-born participants. We also found that mtDNA copy number was positively correlated with the duration of residence in the United States among Mexico-born participants. Even among US-born participants, mtDNA copy number was positively correlated with language acculturation. Although the underlying link between mtDNA copy number and acculturation is unknown, we speculate that the difference may be partially a result of the stresses of immigration and acculturation on the Mexico-born participants. Moreover, immigrants tend to have low socioeconomic status and to live in poor and enclosed neighborhoods. Those factors may translate into financial stress and stress from discrimination. In fact, researchers are increasingly interested in understanding how mitochondrial dysfunction is involved in stress and its related disorders. For example, in a recent study, early-life experiences of adversity and lifetime psychopathology were found to be associated with higher mtDNA copy number (*P* < 0.001) [41]. Similarly, Otsuka *et al.* [42] reported that mtDNA copy number was significantly higher among individuals who committed suicide than in controls.

In the current study, we found that control participants who were overweight had higher mtDNA copy numbers than did those who were obese or who were underweight or normal weight. Similar trend was also reported in prostate cancer patients [43]. Due to the limitation in DNA repair capacity, mitochondria cannot remove or repair DNA damage caused by ROS. To compensate for the damage, healthy mitochondria increase their copy number to counterbalance the metabolic defects in mitochondria carrying mutated mtDNA and the resulting impaired respiratory system [44, 45]. However, mitochondria are the main sites for fatty acid β-oxidation. Loss of mitochondrial enzymes can cause obesity in mice [46]. Thus, our finding might indicate a defect in mitochondrial function due to increased BMI, by which mtDNA copy numbers have to be increased to compensate the loss of healthy mitochondria. However, once BMI reaches above the threshold, mtDNA copy numbers can no longer be increased to cope with the stress caused by fatty acid metabolism. Evidently, more study is wanted to further understand how these factors may influence mtDNA copy number levels.

Additionally, we observed that participants with medium or high levels of physical activity had higher mtDNA copy numbers than did those with low levels of physical activity. Our observation is generally consistent with literature reports. For example, being physically active was found to be associated with higher blood mtDNA copy number in postmenopausal women [47]. Being physical active can increase mitochondrial function in skeletal muscle [48, 49]. Additionally, regular exercise can prevent the ageing process as well as the progression of age-related chronic diseases. While the biological relationships are not fully revealed, anti-oxidative and anti-inflammatory effects are recognized to be major players [48, 50]. In bloods, the mitochondrial transmembrane potential, not only a critical immune system element but also a functional marker of energy status and viability, is affected by exercise [51, 52]. Thus, regular exercise boosts the immune system and reduces inflammatory responses by augmenting blood mitochondrial functions [53].

The major strengths of this study include its large sample size, detailed epidemiologic questionnaire data, and unique Mexican American study population. One weakness of our study is that we were unable to obtain repeated measures of mtDNA copy number because one time quantification may not echo mtDNA copy number over a long period. Lemnrau *et al.* [29] compared mtDNA copy number from blood samples collected approximately 6 years apart and found wide temporal variation. No matched tumor and normal tissues, which would have enabled us to compare mtDNA copy number in target and proxy tissues, were available. While the amount of mtDNA in various cell types is generally consistent, mtDNA copy number shows significant variation by cell type. In addition, there is a possibility that the results from this study may be biased by multiple sources of non-lineage DNA in peripheral blood, such as platelets and cell-free DNAs. Unfortunately, in our study, we don’t have the data on platelet and white blood cell count when the blood was drawn. Finally, we have to be very cautious to generalize the findings from this study to other population. Other cohorts of Mexican Americans are needed to validate our findings. Nevertheless, our study is the first prospectively designed study to show a positive association between blood mtDNA copy number and all-cancer risk. Although blood mtDNA copy number can be affected by various factors, it still have clinical relevance including risk prediction and stratification. Further research is needed to evaluate factors associated with stress, aging, and oxidative damage that influence mtDNA copy number and to define the underlying molecular mechanisms linking blood mtDNA copy number and cancer risk.

## MATERIALS AND METHODS

### Study population

The samples used in this study were obtained from individuals in a large population-based cohort of Mexican-origin households recruited in the Houston, Texas area. Detailed description of the cohort has been illustrated previously [54]. The informed consent was obtained before the interview from each study participant. The incidence of cancer was confirmed with the Texas Cancer Registry. The study was approved by the Institutional Review Board of MD Anderson Cancer Center. All methods were performed in accordance with the relevant guidelines and regulations.

### Determination of mtDNA copy number

Genomic DNA was extracted from whole blood for all the samples by use of QIAamp DNA Mini kits (Qiagen, Valencia, CA). We used a real-time quantitative polymerase chain reaction (PCR) to determine mtDNA copy number. This method, which has high interassay reliability, is detailed in our previous publications [16, 40]. Two pairs of primers were used in 2 steps for relative quantification of mtDNA. One primer pair was used to amplify the mitochondrially encoded NADH:ubiquinone oxidoreductase core subunit 1 (*MTND1*) gene. The other primer pair was used to amplify the single-copy nuclear human globulin (*HGB*) gene. SYBR-green was used in the analysis. The PCRs for two genes were executed on separate 96-well plates with the same samples in the same well positions to avoid possible position effects. A diluted reference DNA, a negative control DNA, and a calibrator DNA were included in each run to generate the standard curves. For each standard curve, the DNA sample was consecutively diluted 1:2 to yield a 7-point standard curve between 0.3125 and 20 ng of DNA. The R^2^ value for each standard curve was 0.99 or higher and the average slope of each standard curve was 3.45. Standard deviations for the threshold cycle (Ct) value were set at 0.25. If the Ct value was higher than this cutoff, the test was repeated.

### Statistical analysis

We used the statistical software package SAS version 9.4 (SAS, Cary, NC) for analysis. Because the mtDNA copy number data were not normally distributed in the control group, we performed the analysis using log-transformed data. First, we evaluated whether mtDNA copy number differed according to selected characteristics in a cancer-free control group. The Student *t* test was used for 2-level dichotomous variables, and analysis of variance was used for variables with more than 2 levels. Next, to further assess the relationships between selected demographic variables and mtDNA copy number in the control group, we performed multivariate unconditional quantile regression analysis. Unconditional quantile regression estimates the effects at different points of the distribution of the endogenous variable, for example at the 10th, 25th, 50th, 75th, and 90th percentiles. This test tells us how the independent variable affects the entire distribution of the dependent variable, not only its mean. Age, sex, marital status, education level, place of birth, body mass index (BMI), alcohol use, and physical activity were included in the unconditional quantile regression model.

To assess relationships between mtDNA copy number and variables measuring acculturation and socioeconomic status in the control group, we performed multivariate linear regression analysis. The model included a language acculturation score; preference for Mexican or US food, friends, social activity, heritage, and holidays; income; home ownership; car ownership; health insurance status; age; sex; and birthplace. For participants born in Mexico, the number of years living in the United States and age of immigration were also included.

Finally, associations among cancer incidence, selected demographic characteristics, and mtDNA copy number were assessed using univariate and multivariable-adjusted Cox proportional hazards regression models. Mitochondrial DNA copy number was analyzed as both a continuous and a categorical variable. For categorical variables, cutoff points were set at the quartile values in the cancer-free control group. Adjusted hazard ratios (HRs) and 95% confidence intervals (CIs) were estimated, and potential confounding factors were adjusted for as appropriate. All statistical tests were 2-sided, and *P* values of less than 0.05 were considered statistically significant.
